# Transient acquisition of cross-species infectivity during the evolution of SARS-CoV-2

**DOI:** 10.1093/nsr/nwab167

**Published:** 2021-09-04

**Authors:** Qi Chen, Xing-Yao Huang, Meng-Xu Sun, Rui-Ting Li, Hongjing Gu, Ying Tian, Rong-Rong Zhang, Dan Luo, Chao Zhou, Yifei Zhang, Tianshu Cao, Na-Na Zhang, Yong-Qiang Deng, Xiao-Feng Li, Cheng-Feng Qin

**Affiliations:** State Key Laboratory of Pathogen and Biosecurity, Beijing Institute of Microbiology and Epidemiology, AMMS, China; State Key Laboratory of Pathogen and Biosecurity, Beijing Institute of Microbiology and Epidemiology, AMMS, China; State Key Laboratory of Pathogen and Biosecurity, Beijing Institute of Microbiology and Epidemiology, AMMS, China; State Key Laboratory of Pathogen and Biosecurity, Beijing Institute of Microbiology and Epidemiology, AMMS, China; State Key Laboratory of Pathogen and Biosecurity, Beijing Institute of Microbiology and Epidemiology, AMMS, China; State Key Laboratory of Pathogen and Biosecurity, Beijing Institute of Microbiology and Epidemiology, AMMS, China; State Key Laboratory of Pathogen and Biosecurity, Beijing Institute of Microbiology and Epidemiology, AMMS, China; State Key Laboratory of Pathogen and Biosecurity, Beijing Institute of Microbiology and Epidemiology, AMMS, China; State Key Laboratory of Pathogen and Biosecurity, Beijing Institute of Microbiology and Epidemiology, AMMS, China; State Key Laboratory of Pathogen and Biosecurity, Beijing Institute of Microbiology and Epidemiology, AMMS, China; State Key Laboratory of Pathogen and Biosecurity, Beijing Institute of Microbiology and Epidemiology, AMMS, China; State Key Laboratory of Pathogen and Biosecurity, Beijing Institute of Microbiology and Epidemiology, AMMS, China; State Key Laboratory of Pathogen and Biosecurity, Beijing Institute of Microbiology and Epidemiology, AMMS, China; State Key Laboratory of Pathogen and Biosecurity, Beijing Institute of Microbiology and Epidemiology, AMMS, China; State Key Laboratory of Pathogen and Biosecurity, Beijing Institute of Microbiology and Epidemiology, AMMS, China; Research Unit of Discovery and Tracing of Natural Focus Diseases, Chinese Academy of Medical Sciences, China

Since the coronavirus disease 2019 (COVID-19) pandemic began, its causative agent, severe acute respiratory syndrome coronavirus 2 (SARS-CoV-2), has spread worldwide. During the global transmission of SARS-CoV-2, mutations in the viral genome have gradually accumulated and have led to the emergence of variants. These emerging variants, including 501Y.V1, 501Y.V2 and 501Y.V3 (also called the alpha, beta and gamma variants, respectively), rapidly became the predominant epidemic strains and subsequently spread worldwide. All three of these SARS-CoV-2 variants contain specific amino acid mutations in the S protein and share an amino acid mutation, N501Y, in the receptor binding domain (RBD) of the S protein (Fig. S1). The RBD specifically binds to the receptor angiotensin-converting enzyme 2 (ACE2) on human cells and mediates host cell entry of SARS-CoV-2.

Interestingly, the N501Y mutation was first documented during *in vivo* passaging of SARS-CoV-2 in mice (Fig. S1), and the resulting mouse-adapted strains MASCp6 and MASCp36 are fully capable of infecting standard laboratory mice [1,2], unlike isolates of the original SARS-CoV-2 strain. Most importantly, we and others have demonstrated that the N501Y mutation significantly enhances the binding affinity of the SARS-CoV-2 RBD for mouse ACE2 [2,3],

thus contributing to the acquired infectivity and pathogenicity phenotype in mice. However, whether naturally occurring SARS-CoV-2 variants (501Y.V1, 501Y.V2 and 501Y.V3) that contain this unique N501Y mutation have acquired the capability to infect mice remains to be determined.

Herein, we adopt a contemporary 501Y.V2 variant, GDPCC, isolated from an imported case in a patient from South Africa to assay infectivity in mice. The SARS-CoV-2 clinical strain IME-BJ05 (wild-type, WT) isolated in the early stage of the COVID-19 pandemic was used as the control strain. Groups of nine-month-old female BALB/c mice were intranasally challenged with the 501Y.V2 variant or WT strain at a dose of 1.2 × 10^4^ pfu. Remarkably, all 501Y.V2-infected mice began to show ruffled fur, hunched posture and reduced activity on day 3 post infection, and significant weight loss was seen in 501Y.V2-infected mice on days 4–6 post infection (Fig. 1A). The 501Y.V2-infected mice finally recovered, and no deaths occurred during the observation period. However, none of the animals challenged with WT virus developed obvious weight loss or clinical symptoms, as expected.

**Figure 1.**
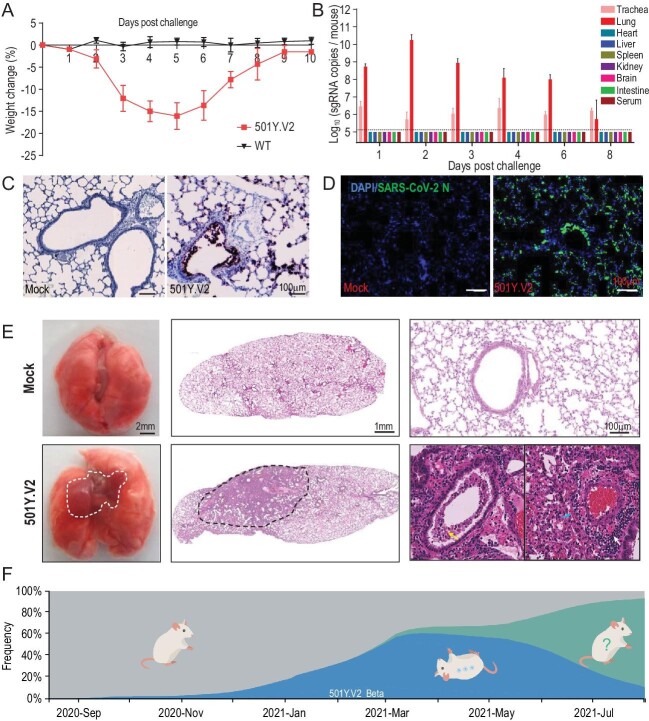
Laboratory mice are susceptible to infection with the SARS-CoV-2 501Y.V2 variant. (A) Body weight changes in nine-month-old female BALB/c mice infected intranasally with 501Y.V2 or IME-BJ05 at a dose of 1.2 × 10^4^ pfu per mouse. *n* = 5. (B) Tissue distribution of SARS-CoV-2 sgRNA. Each tissue and serum sample was subjected to viral sgRNA copy analysis by real-time qPCR. The dotted lines denote the detection limit (*n* = 3). (C) ISH assay for viral RNA in lung tissues from mice infected with 501Y.V2 or treated with PBS (mock) on day 3 post infection. Positive signals are shown in brown. (D) Immunostaining of lung tissues with a SARS-CoV-2 N protein-specific mAb. (E) Gross necropsy and hematoxylin and eosin (H&E) staining of lung tissue sections from mice infected with 501Y.V2 or treated with PBS (mock) on day 3 post infection. (F) Time-resolved frequency distribution of SARS-CoV-2 variants with or without the N501Y mutation (based on the Nextstrain project). The variants with the N501Y mutation are indicated in blue, and mice that can be infected with these variants are shown in an inverted position.

To characterize viral replication dynamics in mice, 501Y.V2- or mock-infected animals were sacrificed, and the major tissues and serum were

collected. SARS-CoV-2 subgenomic RNA (sgRNA) quantitation showed that the highest abundance of viral RNA was detected in lung tissues from 501Y.V2-infected mice, with an obvious increasing trend during the first two days post infection (Fig. 1B). Viral sgRNA remained detectable in the trachea until day 8 post infection (Fig. 1B). However, no detectable sgRNA was present in other tissues or serum. An *in situ* hybridization (ISH) assay with the RNAScope approach showed that viral RNA was located mainly in cells along the airway and at the alveolar walls (Fig. 1C). Immunostaining of lung sections showed that SARS-CoV-2 N protein was expressed mainly in bronchiolar epithelial cells and alveolar cells, consistent with the ISH results (Fig. 1D).

More importantly, gross necropsy showed visible lung injury, characterized by lung enlargement and local perihilar consolidation, upon 501Y.V2 challenge (Fig. 1E, left panel). Microscopic observation of lung sections from 501Y.V2-infected mice also showed that lung injury occurred mainly in the perihilar region (Fig. 1E, middle panel) and was characterized by large quantities of desquamating necrotic epithelial cells in bronchioles (yellow arrow), scattered hemorrhage (blue arrow) and inflammatory cell infiltration within fused alveolar walls (white arrow) (Fig. 1E, right panel). Immunohistochemical staining indicated that the infiltrated inflammatory cells included mainly Neu^+^ neutrophils, CD68^+^ macrophages and CD3^+^ T cells (Fig. S2). Finally, SARS-CoV-2-specific IgG and neutralizing antibodies were detected on days 6 and 8 post infection (Fig. S3). Taken together, these results clearly demonstrate that 501Y.V2 has acquired the capability to replicate in the mouse respiratory tract and to induce typical lung damage and virus-specific immune responses.

Sequence comparison revealed that some emerging SARS-CoV-2 variants harbor the same N501Y mutation as the well-characterized mouse-adapted strains (Fig. S1). Herein, we provide experimental evidence that SARS-CoV-2 501Y.V2 variants are fully capable of infecting standard laboratory mice. After intranasal infection, 501Y.V2 caused significant weight loss in aged mice (Fig. 1A), and the virulence of 501Y.V2 was superior to that of the well-characterized mouse-adapted strain MASCp6 [1], but this variant was less lethal than MASCp36 [2]. Compared with MASCp6, 501Y.V2 carries two additional mutations (K417N and E484K) in the RBD (Fig. S1), and structural and affinity assays have suggested that both the K417N and E484K mutations contribute to the enhanced affinity for mouse ACE2 [4]. A recent study also indicated that the infectivity of pseudotyped SARS-CoV-2 in ACE2-expressing mouse cells harboring the N501Y, K417N and E484K mutations is 4-fold higher than that of SARS-CoV-2 harboring the single N501Y mutation [5]. In addition, MASCp36 harbors triple K417N/Q493H/N501Y mutations in the RBD, which shows higher binding affinity for mouse ACE2 than the RBDs of 501Y.V2 and MASCp6 [2]. Thus, the observed infectivity of 501Y.V2 variants and mouse-adapted strains in mice is closely related to the interaction between the RBD and mouse ACE2. Additionally, we cannot rule out the contribution of other mutations outside the RBD, and further validation with reverse genetic tools is warranted.

Compared with the original SARS-CoV-2 isolates, the 501Y.V2 variant, as well as other variants with the N501Y mutation, has apparently gained the capability to expand its host range to a new species—*Mus musculus*. The experimental results described here are based on laboratory mice and a relatively high challenge dose (1.2 × 10^4^ pfu per mouse), and no evidence of natural transmission of 501Y.V2 in wild mice has been documented to date. However, cats, minks, lions and tigers have been reported to test positive for SARS-CoV-2, and evidence of human-to-animal-to-human transmission of SARS-CoV-2 in mink farms has been reported [6,7]. Thus, molecular and seroepidemiological investigation of SARS-CoV-2 and its variants in wild mice is warranted.

Genomic epidemiology analysis indicates that the presence of the variants harboring the N501Y mutation, including 501Y.V1, 501Y.V2 and 501Y.V3, increased rapidly after their emergence in 2020 and peaked in June 2021, accounting for 62% of all epidemic strains (Fig. 1F). However, the delta variant, which does not carry the N501Y mutation or other known mutations that increase RBD binding to mouse ACE2, emerged suddenly in May 2021 and rapidly became the predominant strain worldwide (Fig. 1F). Currently, the infectivity and pathogenicity of the delta variant in mice remain to be determined. However, the surge and disappearance of SARS-CoV-2 variants with the N501Y mutation suggests that the acquisition of cross-species infectivity in mice is only transient (Fig. 1F). The changing faces of SARS-CoV-2 epidemic strains urge further investigation on the host adaption and natural transmission of SARS-CoV-2 at cross-species interfaces.

Finally, our results also provide a less expensive and more accessible platform for evaluating the *in vivo* efficacy of antivirals and vaccines against SARS-CoV-2. Most previous mouse models of SARS-CoV-2 infection were based on expensive genetically modified mice that express human ACE2 (hACE2) [8–10]; however, standard strains of laboratory mice are readily available in almost any quantity worldwide and are relatively inexpensive. The 501Y.V2-based mouse model described here will open the door for *in vivo* screening and validation of potential COVID-19 therapeutics and vaccine candidates.

## Supplementary Material

nwab167_Supplemental_File
