# Supplementary material for: Transient acquisition of cross-species infectivity during the evolution of SARS-CoV-2
Source: Natl Sci Rev. 2021 Sep 4;8(11):nwab167. doi: 10.1093/nsr/nwab167 (PMC8499877; doi:10.1093/nsr/nwab167)
Supplement: nwab167_Supplemental_File [file nwab167_supplemental_file.docx]

**Supplementary Materials**

**Materials and Methods**

**Bioinformatic analysis**

The time-resolved frequency distribution of SARS-CoV-2 variants was generated with the nextstrain/ncov build maintained by the Nextstrain team using data from GISAID. The distribution diagram showed 3836 representative genomes sampled worldwide between December 2019 and August 2021. A full list of sequence authors is available at nextstrain.org. Full details on bioinformatic processing can be found at https://github.com/nextstrain/ncov.

**Ethics statement**

All procedures involving infectious virus were conducted in the biosafety level 3 (BSL-3) laboratory at the Beijing Institute of Microbiology and Epidemiology, AMMS, and were approved by the Animal Experiment Committee of the Laboratory Animal Center, Beijing Institute of Microbiology and Epidemiology (approval number: IACUC-DWZX-2020-002).

**Mouse and virus strains**

Nine-month-old female BALB/c mice were purchased from Beijing Vital River Laboratory Animal Technology Co., Ltd. The SARS-CoV-2 strain BetaCov/human/CHN/Beijing_IME-BJ05/2020 (IME-BJ05, accession no. GWHACAX01000000) was originally isolated from COVID-19 patients and propagated in Vero cells. The 501Y.V2 variant GDPCC (CSTR: 16698.06.NPRC2.062100001) was isolated from an imported case in a patient from South Africa and stored at the National Pathogen Resource Center (NPRC), China. Both viruses were propagated and titrated by a standard plaque assay in Vero cells.

**Mouse challenge experiments**

For intranasal infection, mice were anesthetized with sodium pentobarbital at a dose of 50 mg/kg via the intraperitoneal route and were then subjected to intranasal instillation of 1.2×10^4^ pfu of 501Y.V2, 1.2×10^4^ pfu of IME-BJ05 (WT) or PBS (mock). Mice were then weighed and monitored daily and were sacrificed at the indicated time points for serum collection and tissue processing.

**Measurement of viral sgRNA**

Tissue homogenates were clarified by centrifugation at 6,000 rpm for 6 minutes, and the supernatants were transferred to a new Eppendorf (EP) tube. RNA was extracted using a QIAamp Viral RNA Mini Kit (Qiagen) according to the manufacturer’s protocol. sgRNA was quantified in each sample by reverse transcription–quantitative PCR (RT-qPCR) with primers targeting the S gene of SARS-CoV-2. RT-qPCR was performed using a One Step PrimeScript RT-PCR Kit (Takara) with the following primers and probes: sgRNA-F (5’-CGATCTCTTGTAGATCTGTTCTC-3’), sgRNA-R (5’-ATATTGCAGCAGTACGCACACA-3’), and sgRNA-P3 (5’-ACACTAGCCATCCTTACTGCGCTTCG-3’).

**RNA ISH assay**

The RNA ISH assay of the SARS-CoV-2 genome was performed with an RNAScope 2.5 HD Reagent Kit (Advanced Cell Diagnostics) according to the manufacturer’s instructions. Lung tissues were fixed with 4% PFA for 48 hours and embedded in paraffin in accordance with the standard procedure. The formalin-fixed, paraffin-embedded tissue sections (4 μm) were deparaffinized by incubation for 60 minutes at 60°C. Endogenous peroxidase activity was quenched with hydrogen peroxide for 10 minutes at room temperature. Sections were then boiled for 15 minutes in RNAScope Target Retrieval Reagents and incubated for 30 minutes in RNAScope Protease Plus before probe hybridization. Tissues were counterstained with Gill’s hematoxylin and visualized by standard bright-field microscopy.

**Histopathological analysis**

Paraffin tissue sections (4 μm thick) were deparaffinized with xylene, rehydrated through successive ethanol/water baths and washed with distilled water at room temperature. The deparaffinized sections were stained with H&E and examined by light microscopy. Lung tissue lesions were assessed mainly according to the degeneration and necrosis of bronchiolar epithelial cells and alveolar pneumocytes, changes in alveolar structure, infiltration of inflammatory cells and presence of hemorrhage.

**Immunofluorescence staining**

For immunostaining, paraffin tissue sections were deparaffinized as described above and were then put in EDTA (pH 9.0) for 1 hour at 96°C for antigen retrieval. Endogenous peroxidases were inactivated with 3% hydrogen peroxide for 25 minutes, and the sections were then blocked with 3% BSA for 30 minutes. SARS-CoV/SARS-CoV-2 Nucleocapsid Antibody, Mouse mAb (Sino Biological, 1:500) was used as the primary antibody and incubated with the sections for 2 hours in a humidified chamber at 37°C. After 3 washes, the sections were incubated with a FITC-labeled secondary antibody (Zhongshan Biotechnology) for 1 hour prior to DAPI staining for 5 minutes.

**Immunohistochemical staining**

To detect inflammatory cell infiltration, paraffin tissue sections were treated as described above and were then incubated with a rabbit anti-LY6G polyclonal antibody (Servicebio, 1:800), rabbit anti-CD68 polyclonal antibody (Servicebio, 1:200) or rabbit anti-CD3 polyclonal antibody (Servicebio, 1:200) overnight at 4°C. After three washes, the sections were incubated with a horseradish peroxidase (HRP)-conjugated secondary antibody (Servicebio, 1:200) at 37°C for 1 hour prior to DAB staining (Servicebio). The sections were counterstained with hematoxylin for observation by microscopy.

**ELISA**

SARS-CoV-2 S-specific IgG titers were determined with a commercial ELISA kit (Beijing Wantai Biological) according to the manufacturer’s instructions. In brief, serial 2-fold dilutions (starting at 1:10) of inactivated serum were added to 96-well plates (50 μl/well) coated with recombinant SARS-CoV-2 S antigen and incubated for 30 minutes at 37°C. After three washes with wash buffer, HRP-conjugated goat anti-mouse IgG (1:5,000, ZSGB-BIO) was added to the plates and incubated for 30 minutes at 37°C. After washing the plates five times with wash buffer, chromogen solution was added for 15 minutes of incubation at 37°C. After the addition of stop solution, the absorbance (450/630 nm) was read using a microplate reader (Bio Tek). The endpoint titers were defined according to the manufacturer’s instructions.

**Microneutralization assay**

The SARS-CoV-2-specific neutralizing antibody titer in serum was determined using a cytopathic effect (CPE)-based microneutralization assay with 501Y.V2 variant. In brief, serum samples were heat inactivated for 30 minutes at 56°C and serially diluted 2-fold from 1:10 to 1:160 using DMEM (Thermo Fisher Scientific). Serum dilutions (50 μl) were then mixed with the same volume of virus solution containing 100 median tissue culture infectious dose (TCID_50_) of SARS-CoV-2. The serum-virus mixture was incubated at 37°C for 1 hour and was then added to 96-well plates containing semiconfluent Vero cells (ATCC, CCL-81, >80% density). After culture at 37°C for 3 days, the CPE was assessed under an inverted microscope. The neutralization titer was calculated as the reciprocal of the highest sample dilution that protected 50% of the wells from CPE.

**Statistical analysis**

Statistical analyses were carried out using Prism software (GraphPad Prism 7.0). In all experiments, the data are presented as the mean ± SEM values. Statistical details of the experiments and numbers of replicate animals (n) are stated in the relevant figure legends and method details.


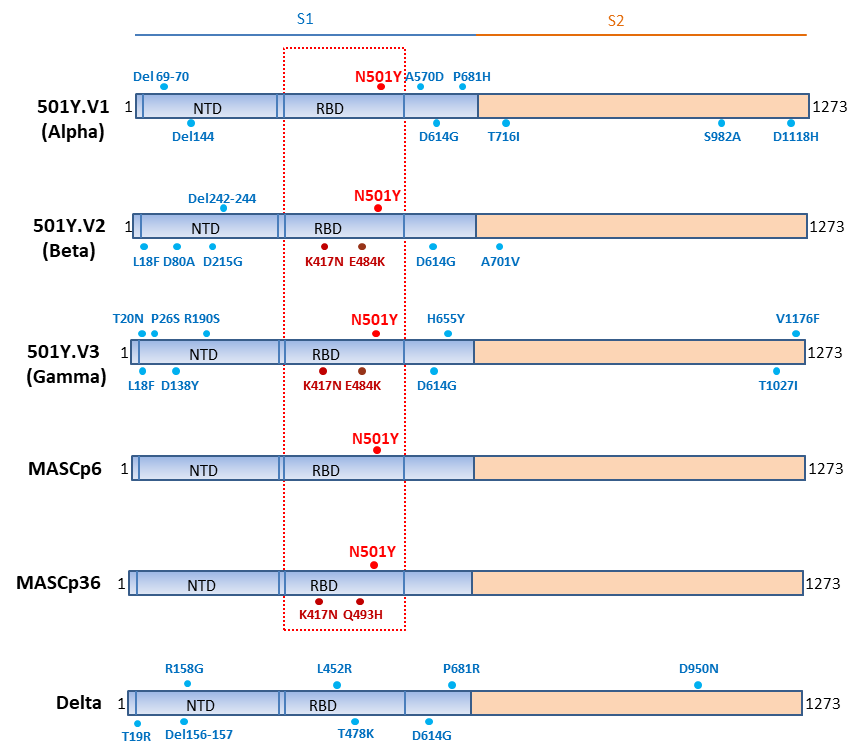


**Figure S1.** Illustration of the amino acid mutations in the S protein of 501Y.V1, 501Y.V2, 501Y.V3, delta, MASCp6 and MASCp36 compared with WT SARS-CoV-2.


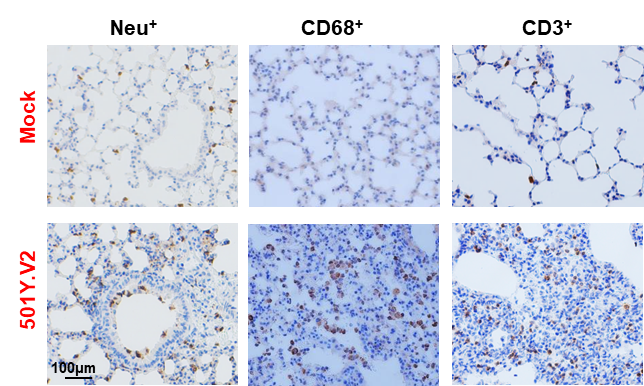


**Figure S2.** IHC analysis of neutrophils (Neu^+^) (A), macrophages (CD68^+^) (B) and T cells (CD3^+^) in lung tissues from mice infected with 501Y.V2 or treated with PBS (mock) on day 3 post infection.


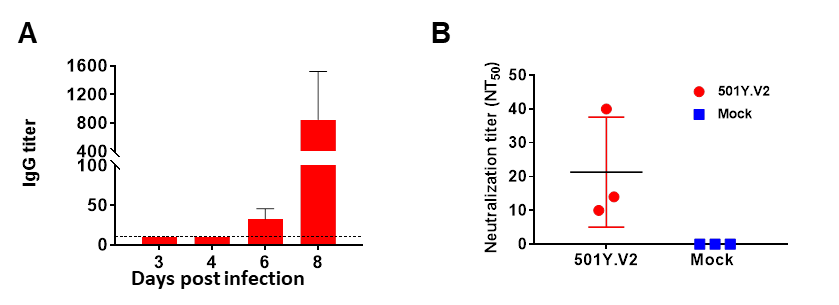


**Figure S3.** Humoral immune response in 501Y.V2-infected mice. Nine-month-old female BALB/c mice were infected intranasally with the 501Y.V2 variant at a dose of 1.2×10^4^ pfu per mouse. (A) ELISA for SARS-CoV-2 S-specific IgG antibodies in sera collected on days 3, 4, 6 and 8 post infection (n=3). The dotted line indicates the detection limit of the assay. (B) Microneutralization assay of sera from mice infected with 501Y.V2 or treated with PBS (mock) on day 8 post infection (n=3).
